# Predicting the Epidemic Sizes of Influenza A/H1N1, A/H3N2, and B: A Statistical Method

**DOI:** 10.1371/journal.pmed.1001051

**Published:** 2011-07-05

**Authors:** Edward Goldstein, Sarah Cobey, Saki Takahashi, Joel C. Miller, Marc Lipsitch

**Affiliations:** 1 Center for Communicable Disease Dynamics, Department of Epidemiology, Harvard School of Public Health, Boston, Massachusetts, United States of America; 2 Department of Applied Mathematics, Harvard University, Cambridge, Massachusetts, United States of America; 3 Fogarty International Center, US National Institutes of Health, Bethesda, Maryland, United States of America; 4 Department of Immunology and Infectious Diseases, Harvard School of Public Health, Boston, Massachusetts, United States of America; Johns Hopkins University, United States of America

## Abstract

**Background:**

The epidemic sizes of influenza A/H3N2, A/H1N1, and B infections vary from year to year in the United States. We use publicly available US Centers for Disease Control (CDC) influenza surveillance data between 1997 and 2009 to study the temporal dynamics of influenza over this period.

**Methods and Findings:**

Regional outpatient surveillance data on influenza-like illness (ILI) and virologic surveillance data were combined to define a weekly proxy for the incidence of each strain in the United States. All strains exhibited a negative association between their cumulative incidence proxy (CIP) for the whole season (from calendar week 40 of each year to calendar week 20 of the next year) and the CIP of the other two strains (the complementary CIP) from the start of the season up to calendar week 2 (or 3, 4, or 5) of the next year. We introduce a method to predict a particular strain's CIP for the whole season by following the incidence of each strain from the start of the season until either the CIP of the chosen strain or its complementary CIP exceed certain thresholds. The method yielded accurate predictions, which generally occurred within a few weeks of the peak of incidence of the chosen strain, sometimes after that peak. For the largest seasons in the data, which were dominated by A/H3N2, prediction of A/H3N2 incidence always occurred at least several weeks in advance of the peak.

**Conclusion:**

Early circulation of one influenza strain is associated with a reduced total incidence of the other strains, consistent with the presence of interference between subtypes. Routine ILI and virologic surveillance data can be combined using this new method to predict the relative size of each influenza strain's epidemic by following the change in incidence of a given strain in the context of the incidence of cocirculating strains.

*Please see later in the article for the Editors' Summary*

## Abstract 2

Using weekly influenza surveillance data from the US CDC, Edward Goldstein and colleagues develop a statistical method to predict the sizes of epidemics caused by seasonal influenza strains. This method could inform decisions about the most appropriate vaccines or drugs needed early in the influenza season.

## Editors' Summary

**Background:**

Every winter in temperate countries, millions of people catch influenza, a viral infection of the nose, throat, and airways. Most infected individuals recover quickly but seasonal influenza outbreaks (epidemics) kill about half a million people annually. Epidemics of influenza occur because small but frequent changes in the viral proteins (antigens) to which the immune system responds mean that an immune response produced one year provides only partial protection against influenza the next year. Annual immunization with a vaccine that contains killed influenza viruses of the major circulating strains boosts this natural immunity and greatly reduces a person's chances of catching influenza. Influenza epidemics in temperate latitudes are usually caused by an influenza B virus or one of two influenza A subtypes called A/H3N2 and A/H1N1. The names of the influenza A viruses indicate the types of two major influenza antigens—hemagglutinin (H3 or H1) and neuraminidase (N2 or N1)—present in the viruses.

**Why Was This Study Done?:**

At present, there is no way to predict whether influenza B or an influenza A subtype will be dominant (responsible for the majority of infections) in any given influenza season. There is also no way to predict the size of the epidemic that will be caused by each viral strain. Public health officials would like to be able to make predictions of this sort early in the winter to help them determine which measures to recommend to minimize the illness and death caused by influenza. In this study, the researchers use weekly influenza surveillance data collected by the US Centers for Disease Control and Prevention (CDC) to study the temporal dynamics of seasonal influenza in the United States between 1997 and 2009 and to develop a statistical method to predict the sizes of epidemics caused by influenza A/H1N1, A/H3N2, and B.

**What Did the Researchers Do and Find?:**

The CDC influenza surveillance system collects information on the proportion of patients attending US outpatient facilities who have an influenza-like illness (fever and a cough and/or a sore throat in the absence of any known cause other than influenza) and on the proportion of respiratory viral isolates testing positive for specific influenza strains at US viral surveillance laboratories. The researchers combined these data to define a weekly “proxy” incidence of each influenza strain across the United States (an estimate of the number of new cases per week in the US population) and a cumulative incidence proxy (CIP) for each influenza season. For each strain, there was a negative association between its whole-season CIP and the early-season CIP of the other two strains (the complementary CIP). That is, high infection rates with one strain appeared to interfere with the transmission of other strains. Given this relationship, the researchers then developed a statistical algorithm (a step-by-step problem solving method) that accurately predicted the whole-season CIP for a particular strain by following the incidence of each strain from the start of the season until either its CIP or the complementary CIP had exceeded a specific threshold. So, for example, for influenza B, the algorithm provided an accurate prediction of the whole-season CIP before the peak of influenza B incidence for each season included in the study. Similarly, prediction of whole-season A/H3N2 incidence always occurred several weeks in advance of its weekly incidence peak.

**What Do These Findings Mean?:**

These findings suggest that early circulation of one influenza strain is associated with a reduced total incidence of other strains, possibly because of cross-subtype immunity. Importantly, they also suggest that routine early-season surveillance data can be used to predict the relative size of the epidemics caused by each influenza strain in the United States and in other countries where sufficient surveillance data are available. Because the algorithm makes many assumptions and simplifies the behavior of influenza epidemics, its predictions may not always be accurate. Moreover, it needs to be tested with data collected over more influenza seasons. Nevertheless, the algorithm's ability to predict the relative epidemic size of A/H3N2, the influenza strain with the highest death rates, several weeks before its peak in seasons in which it was the dominant strain suggests that this predictive method could help public-health officials introduce relevant preventative and/or treatment measures early in each influenza season.

**Additional Information:**

Please access these Web sites via the online version of this summary at http://dx.doi.org/10.1371/journal.pmed.1001051.

- The US Centers for Disease Control and Prevention provides information for patients and health professionals on all aspects of seasonal influenza, including information about the US influenza surveillance system
- The UK National Health Service Choices Web site also provides information for patients about seasonal influenza; the UK Health Protection Agency provides information on influenza surveillance in the UK
- MedlinePlus has links to further information about influenza l (in English and Spanish)

## Introduction

Influenza epidemics in temperate latitudes are usually characterized by the dominance of influenza B or one of two influenza A subtypes, A/H3N2 and A/H1N1. There are currently no formal methods to predict which type or subtype (henceforth referred to in this article as “strain”) will dominate in a given season or what the epidemic size of each strain might be. This paper examines how routine surveillance data collected during the course of an influenza season can be used to make predictions for that season. We also investigate whether there is support for the hypothesis that the dynamics of different strains are interdependent, and specifically whether high incidence of one strain interferes with the circulation of other strains within a season.

Previous epidemiological studies have suggested that subtypes of influenza A, and potentially influenza A and influenza B, might compete with each other. Sonoguchi and colleagues [1] studied the impact of the same-season circulation of A/H3N2 and A/H1N1 influenza in Japanese schools and concluded that infection with A/H3N2 was negatively associated with subsequent infection with A/H1N1. Cowling et al. [2] have found that those infected with seasonal influenza A during the 2008–2009 season in Hong Kong had a lower risk of laboratory-confirmed pandemic A/H1N1 infection. Ferguson et al. [3] and Tria et al. [4] concluded that strong, transient, nonspecific immunity effective against all influenza strains was necessary to produce realistic patterns of sequence diversity in simulations of influenza A and B evolution. Cobey and colleagues (personal communication) found evidence of cross-immunity between influenza A/H3N2, A/H1N1, and B in St. Petersburg, Russia. Skowronski et al. [5] found that people vaccinated against seasonal influenza in 2008–2009 were more likely to get infected in the subsequent wave of A/pandemic (H1N1) 2009 (H1N1pdm) compared to individuals who have not received seasonal influenza vaccination, an effect that may have been caused in part by reduced risk of seasonal influenza infection (owing to receipt of seasonal influenza vaccine), and hence reduced cross-subtype immunity.

In this study, US Centers for Disease Control (CDC) data between 1997 and 2009 retrieved from a publicly available database [6] were examined for epidemiological evidence of interaction in the dynamics of the three influenza strains that circulated prior to the 2009 pandemic, A/H3N2, A/H1N1, and B. We utilized two components of the CDC influenza surveillance system: virologic surveillance and outpatient ILI surveillance, described in the Methods and in more detail in [7]. For each season, we defined a proxy for strain-specific incidence (henceforth simply “incidence”) of each strain and defined the cumulative incidence proxy (CIP) for any strain at a given time as the sum of its incidence proxy since the start of the season—see the first section of the Methods for the precise definition and the discussion of its potential limitations. For every chosen (index) strain, the “complementary” CIP was defined as the sum of the CIPs of the other two strains. The index strain's CIP for the whole season and the complementary CIP early in the season were found to be negatively correlated. In particular, for each index strain, the seasons with the largest early complementary CIP were also the seasons with the smallest whole-season CIP, which is a proxy of the strain's epidemic size. This finding naturally leads to two questions: First, is there a threshold for the complementary CIP that predicts a small epidemic for the index strain? Second, if this complementary CIP threshold isn't reached, how does one predict the epidemic size of the index strain? This paper introduces a method for making these predictions. The approach is to follow the incidence of each strain from the start of a season until either the CIP of the index strain or the complementary CIP surpass certain thresholds that are delineated in this study. Once the threshold is reached, the CIP of the index strain for the whole season is predicted in terms of its recent incidence and the time that the threshold is reached. Historical data [6] were used to estimate the prediction parameters as well as the accuracy and timing of predictions.

## Methods

### Strain-Specific Incidence

Data from [6] for the 12 influenza seasons from 1997–1998 to 2008–2009 were used in the analysis. These data are based on two components of the CDC influenza surveillance system: virologic surveillance and outpatient ILI surveillance [7]. Virologic surveillance utilizes data from approximately 80 U.S. World Health Organization (WHO) Collaborating Laboratories and 60 National Respiratory and Enteric Virus Surveillance System (NREVSS) laboratories (which are mostly hospital laboratories) located throughout the United States. The U.S. WHO and NREVSS collaborating laboratories report the total number of respiratory specimens tested and the number positive for influenza types A and B each week. Most of the U.S. WHO collaborating laboratories also report the influenza A subtype (H1 or H3) of the viruses they have isolated. For the ILI surveillance network, each week approximately 1,800 outpatient care sites around the country report data on the total number of patients seen and the number of those patients with ILI by age group. For this system, ILI is defined as fever (temperature of 37.8°C or greater) and a cough and/or a sore throat in the absence of a known cause other than influenza.

Each influenza season was defined as the time between week 40 of one calendar year (epidemiological week 1 in our notation) and week 20 of the next calendar year (epidemiological week 33). The emergence of pandemic A/H1N1 in the 2008–2009 season changed testing and reporting patterns starting in calendar week 17. Therefore, the ending time of the 2008–2009 season was defined to be calendar week 16 of 2009.

We defined a proxy of weekly strain-specific incidence in the United States. For each of the ten regions defined by the US Department of Health and Human Services [6], the proxy for incidence was defined as the product of the proportion of ILI among all outpatient visits to sentinel physicians and the proportion of respiratory viral isolates tested that were positive for a particular strain [6]. For some influenza A isolates, subtyping was not performed. For those samples, the share of A/H1N1 and A/H3N2 was assumed to be proportional to their share among the subtyped strains. The national proxy for incidence was calculated as the sum of the regional proxies weighted by the regions' population sizes. The size of the regional population for each season between calendar years *X* and *X*+1 was estimated as the mean of the regional population estimates for July 1 for those years from census data [8].

This proxy would be a perfect measure (up to a multiplicative constant) of the incidence of infection with a particular strain if the following conditions were met: (i) the fraction of ILIs that result in medical consultations, while possibly varying by strain, does not vary by year or by week; (ii) the numbers of physician visits for all causes in the sentinel practices were consistent from week to week during the season; (iii) the sentinel practices were representative of the full population; and (iv) viral testing, which is conducted in a separate system, was performed on isolates representative of those obtained from patients consulting the sentinel practices for ILI. In reality, none of these assumptions is fully accurate; in particular, samples are selected for testing on the basis of severity of ILI, as well as a part of routine sampling. Nonetheless, we take this proxy measure as the best relative measure of influenza strain-specific incidence that can be calculated from surveillance data.

### Association between the Early Activity of Other Strains and the Total Activity of Each Strain of Interest

To measure patterns in the observations, for each index strain, the complementary CIP up to each of several possible calendar weeks was compared to the CIP of the index strain for the entire influenza season. We examine the Spearman rank correlation between those pairs of numbers (i.e., complementary CIP up to a defined week and the whole season CIP of the index strain) for each calendar week for the 12 seasons in the data.

If circulation of each strain results in interference with the others, then such a negative correlation could arise by two mechanisms. First, high complementary incidence could slow the spread of the index strain. Second, large seasons of an index strain may begin early, interfering with the spread of the complementary strains by the chosen calendar week. We attempt in Text S1 to disentangle these two mechanisms.

### Prediction Model for Cumulative Incidence of a Strain

Given the observed inverse association between the early complementary CIP and the whole-season CIP of each index strain (described in the early part of the Results section, below), we hypothesized that it would be possible to create a prediction algorithm for the whole-season CIP of each strain. We considered a model in which a prediction is triggered when the CIP of either the index strain or the complementary strains reaches a particular threshold. The week at which this threshold is crossed naturally varies from season to season, and the thresholds for the index strain and the complementary strains are allowed to differ. The first time a threshold is reached, the whole-season CIP of the index strain is predicted linearly in terms of a proxy *X* of the index strain's recent growth rate as well as the (weighted) time *T* of crossing the threshold.

More precisely, one follows the incidence of each strain in time from the start of a season until either the cumulative incidence of the index strain in the previous 5 wk surpasses a certain threshold *h* or the complementary CIP from the start of a season surpasses a certain threshold *h*_c_ (both *h* and *h*_c_ vary by index strain as the different strains have quite different distributions of epidemic sizes). The time at which one of these conditions is first met is the “stopping time” *s*. At time *s*, the whole-season CIP of the index strain is predicted. Denoting the index strain's incidence in week *w* as *I*(*w*), the proxy for recent growth is
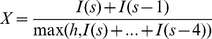
(1)


To elucidate the meaning of the predictor in equation (1), note that if the index strain's CIP reaches its threshold, *h*, first (before the complementary CIP reaches its threshold, *h*_c_) during a period of positive growth, then *X* is related to the recent growth rate of the index strain. If the index strain's CIP reaches its threshold first but during a period of no growth or decline, both the covariate *X* and index strain's whole-season CIP, which is the outcome *Y*, are expected to be smaller compared to the former scenario. If the complementary CIP reaches its threshold first, both the covariate *X* and the outcome *Y* are expected to be smaller compared to the two scenarios above.

For influenza B and A/H1N1, thresholds can be chosen so that the covariate *X* is highly correlated with the outcome *Y* and the linear regression of *Y* against *X* has no outliers (Text S1, section 8). However for influenza A/H3N2, the 2003–2004 season was especially large and early and is an outlier for such regression. That season was driven by the antigenically novel A/Fujian-like strain, which presumably had a larger pool of susceptibles than previous A/H3N2 strains, ensuring an early, strong season. In particular the threshold for the 2003–2004 season was crossed on calendar week 46—4 wk earlier than in any other season. The rate of transmission of a strain of influenza is affected not only by its antigenic novelty but also by seasonal forcing [9],[10]. Correspondingly, the covariate *X* on week 46 in 2003, being a function of the incidence's growth rate at that time, underestimates the cumulative size of A/H3N2 incidence during that year relative to other years. Similarly, for the 2010–2011 season, the proxy of influenza B incidence crossed its threshold exceptionally early compared to the 1997–2009 seasons, and prediction based on the covariate *X* alone is expected to significantly underestimate the whole-season CIP for influenza B for 2010–2011.

To adjust for the above phenomenon of an early crossing of the index strain's threshold *h*, we tested a set of models containing an additional predictor *T* to help explain how the threshold *h* relates to a strain's epidemic size. As described in Text S1, the best-performing model in this set uses a definition of *T* that differs for A/H3N2 versus A/H1N1 and influenza B. For A/H3N2, *T* equals the week *s* in which the threshold is crossed. For A/H1N1 and influenza B, *T* equals the week *s* in which the threshold is crossed, unless the index strain's season is small. The season is defined to be “small” if by week *s*, the cumulative incidence of the index strain is less than *h*; in this case, the covariate *T* is set to 0. For each strain, the predictor *T* is negatively associated with the outcome *Y*, reflecting the fact that an index strain that crosses its threshold *h* early in the season will have a larger epidemic. To understand the motivation behind this definition of *T*, we note that for A/H3N2, seasons in which the complementary threshold is crossed first are generally associated with later stopping times, and the week *s* ( = *T*) of crossing is negatively correlated with the epidemic size of A/H3N2 (outcome *Y*). For A/H1N1 and B, a high complementary CIP early in the season implies that the complementary threshold was crossed before the CIP threshold of the index strain, and the timing of such crossing is positively correlated with the outcome *Y*. For such seasons, which, in addition, have “small” CIP of the index strain since the beginning of the season (as defined above), the covariate *T* is set to 0.

With the covariates *X* and *T* defined above, the CIP *Y* is estimated by
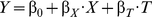
(2)The coefficients in the linear prediction above were estimated by ordinary least squares from the historical data in [6]. For influenza A/H1N1 and B, the intercept β_0_ was not found to be statistically significant. This result is not surprising, given that for strong complementary seasons for those strains, both the outcome and the covariates are quite small. This intercept was therefore not used in the linear regression.

A basic measure of the accuracy of the prediction given by equation (2) is the residual standard error (RSE)
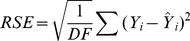
(3)Here *Y*_i_ are the observed outcomes, 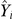 are the predicted outcomes and *DF* is the number of degrees of freedom. We chose the thresholds *h* and *h*_c_ for each strain by visually inspecting the RSEs for a wide range of thresholds (Text S1, figure S6) and determining the space of thresholds where RSE is low and stable.

### Sensitivity Analysis

We performed extensive sensitivity analyses on the parameters used for prediction and the associations between the CIPs of various strains (Text S1).

## Results

### Trends in Incidence and the Association between the Epidemic Size of an Index Strain and the Early Complementary Cumulative Incidence

**Figure 1:**
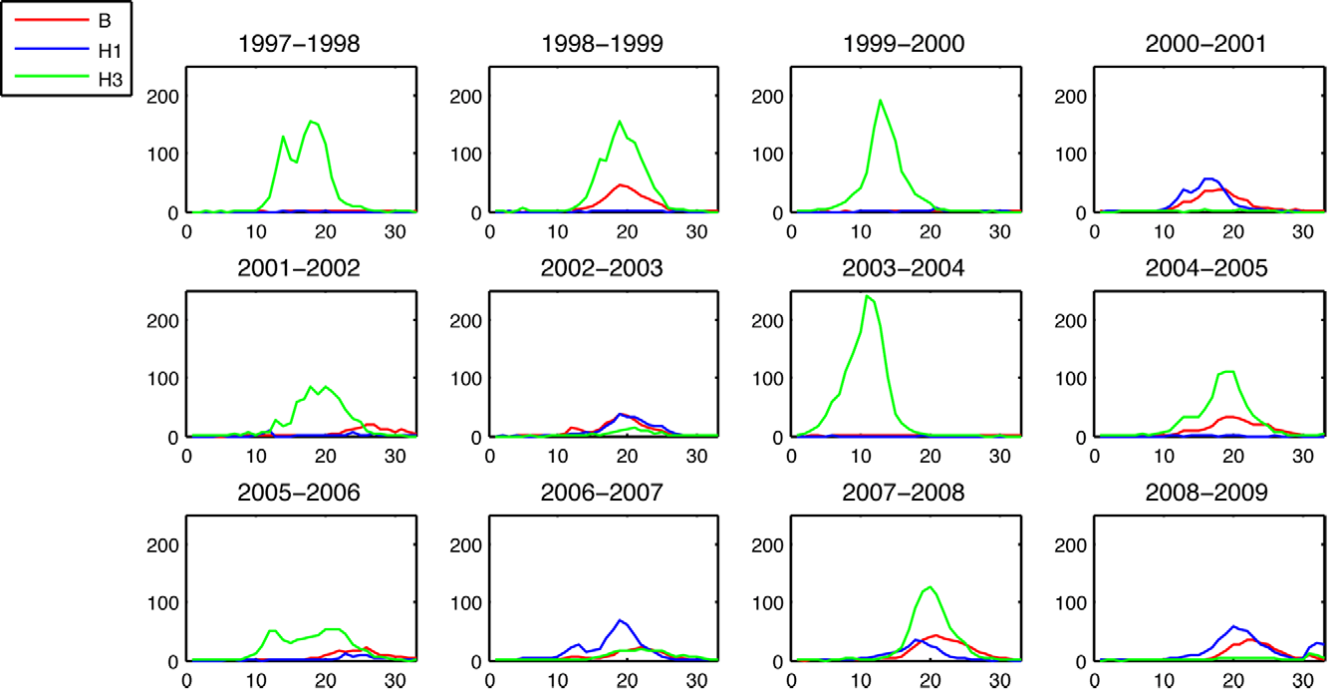
Weekly incidence proxies of A/H3N2 (green), A/H1N1 (blue), and influenza B (red) strains inferred from [**6**].

The weekly incidence proxies of each strain show clear differences from season to season (Figure 1). In most seasons, A/H3N2 has the highest incidence proxy in every week, and B and A/H1N1 were comparatively scarce. In three seasons, the trend is partially reversed: A/H1N1 and B are more prevalent than A/H3N2 in 2000–2001, 2002–2003, and 2008–2009. In 2006–2007, the incidence of A/H1N1 was distinctly higher than that of A/H3N2 and B.

**Figure 2:**
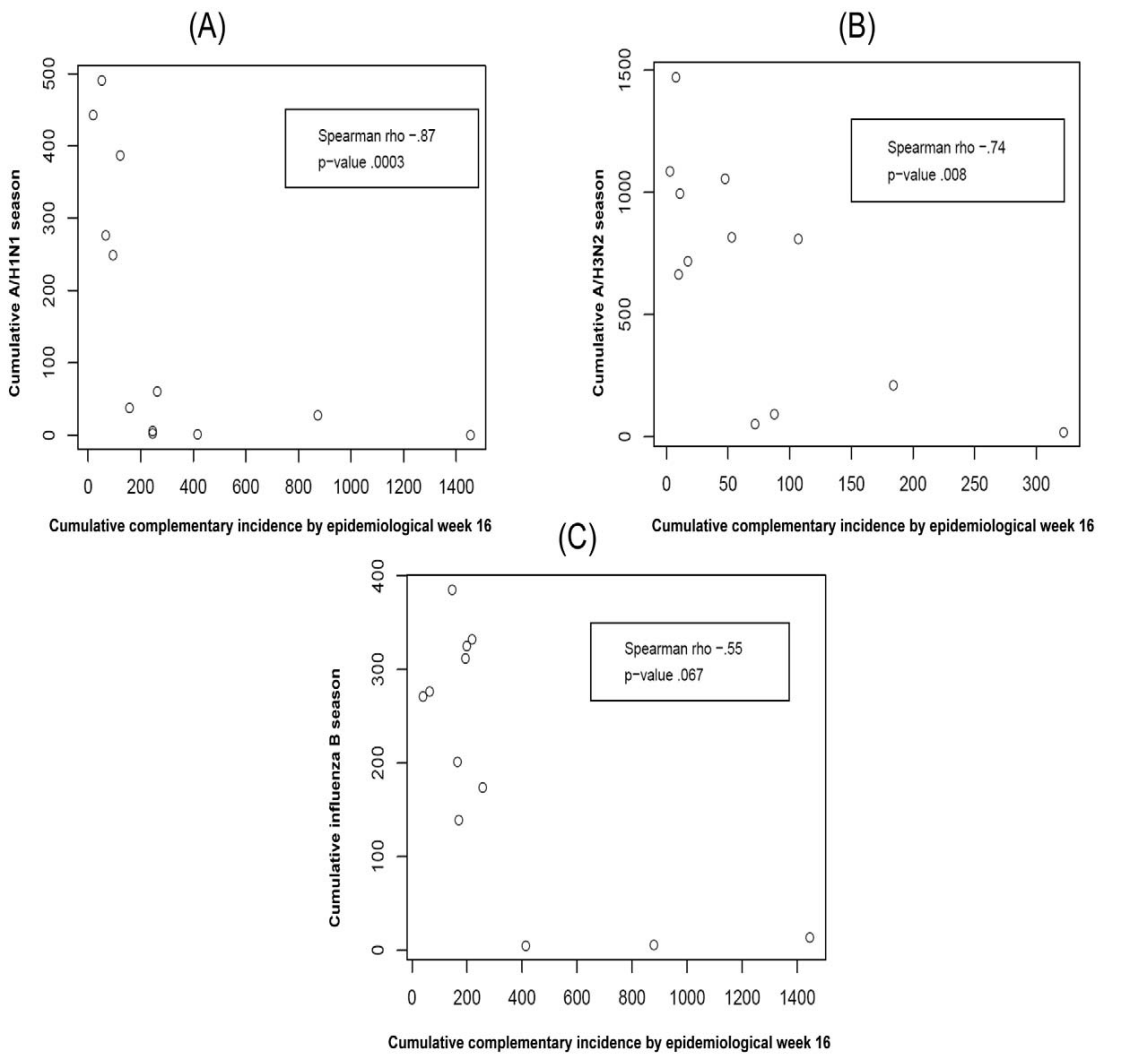
The relationship between the cumulative complementary incidence for each of the index strains A/H1N1 (A), A/H3N2 (B), and B (C) by epidemiological week 16 (calendar week 3) and the index strain's cumulative incidence over the entire season (i.e., its epidemic size) for the 12 y in the data.

There is a negative correlation between the complementary CIP early in the season and the whole-season CIP of the index strain. This relation is significant for index strains A/H1N1 and A/H3N2 for each of the calendar weeks 2, 3, 4, and 5; for index strain B, this relation is significant only for calendar week 2. The relation between the complementary CIP by calendar week 3 and the index strain's whole-season CIP is plotted in Figure 2.

Though the association for influenza B was not statistically significant the three seasons in which complementary CIP was highest for B had the three smallest whole-season CIP for B.

### Prediction of the Epidemic Size of Each Strain

This section presents prediction results for a choice of thresholds for each index strain.

**Figure 3:**
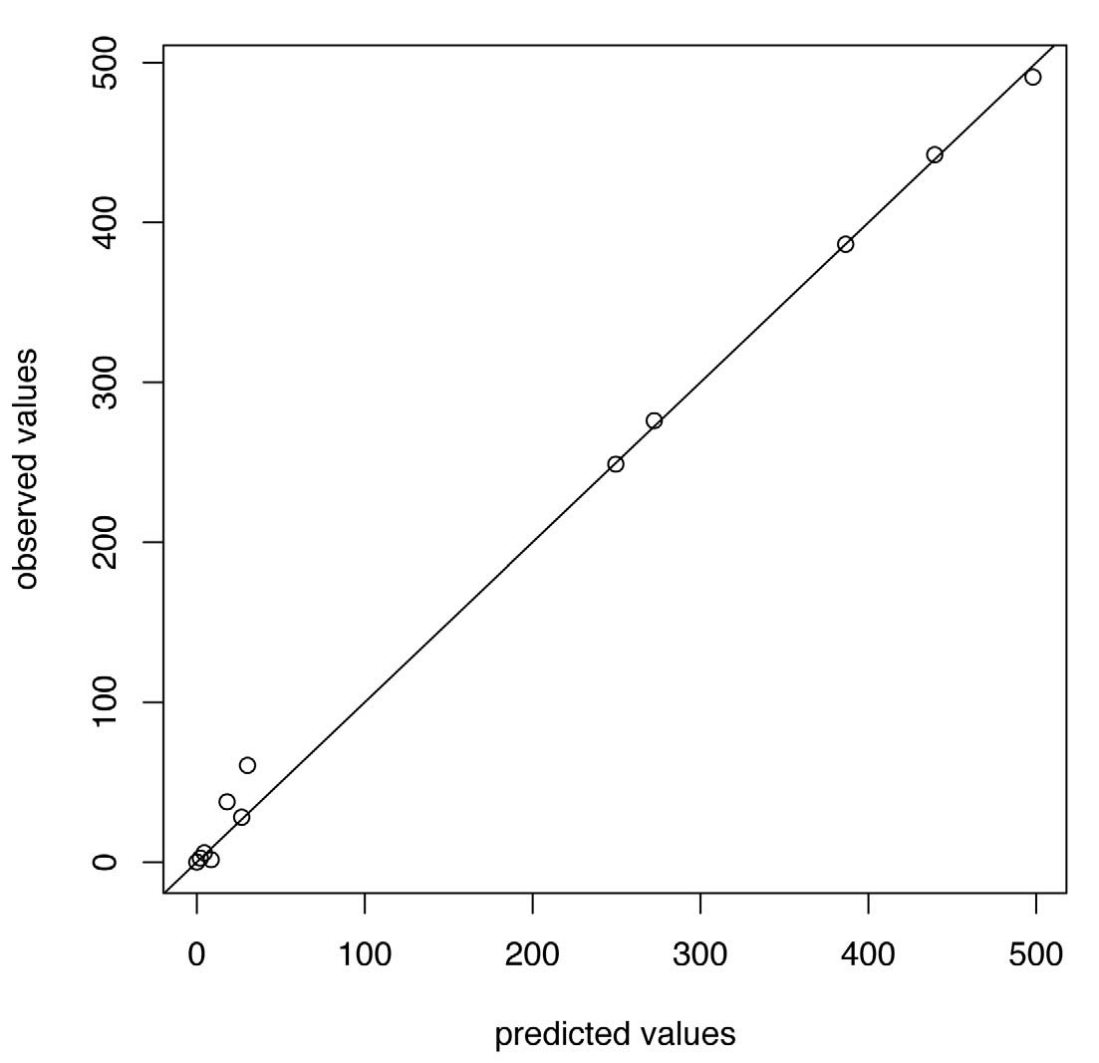
Predicted versus observed values for influenza A/H1N1 for the choice of thresholds *h* = 140, *h*_c_ = 500.

**Figure 4:**
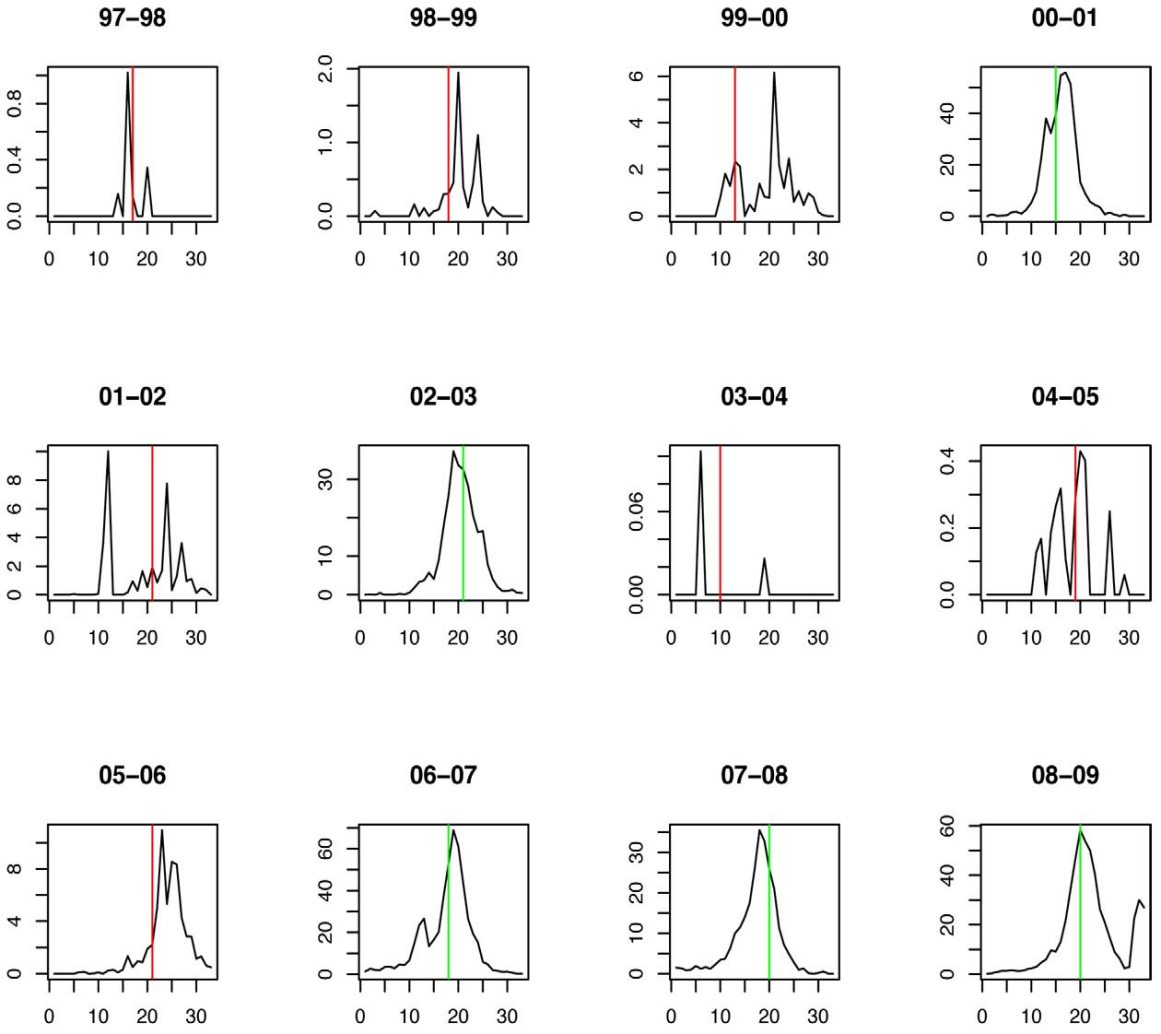
Timing of prediction with regard to weekly influenza A/H1N1 incidence (week 1 = calendar week 40). Green lines show the stopping time *s* in seasons in which the index strain's own threshold was crossed first; red lines indicate the stopping times in seasons in which the complementary threshold was crossed first.

For A/H1N1 the chosen thresholds are *h* = 140, *h*_c_ = 500 (Figures 3 and 4). In the five seasons with appreciable A/H1N1 (i.e., a peak incidence exceeding 30), the prediction occurred before the peak in two seasons, at the peak in one, and following the peak in the remaining two.

**Figure 5:**
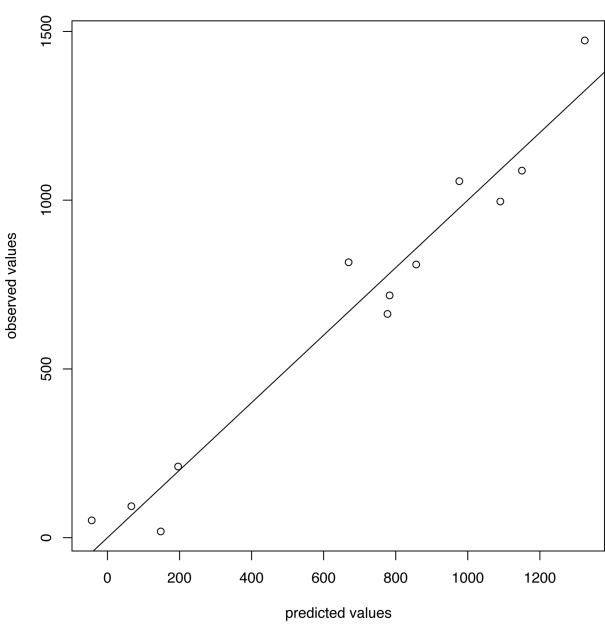
Predicted versus observed values for influenza A/H3N2 for the choice of thresholds *h* = 165, *h*_c_ = 350.

**Figure 6:**
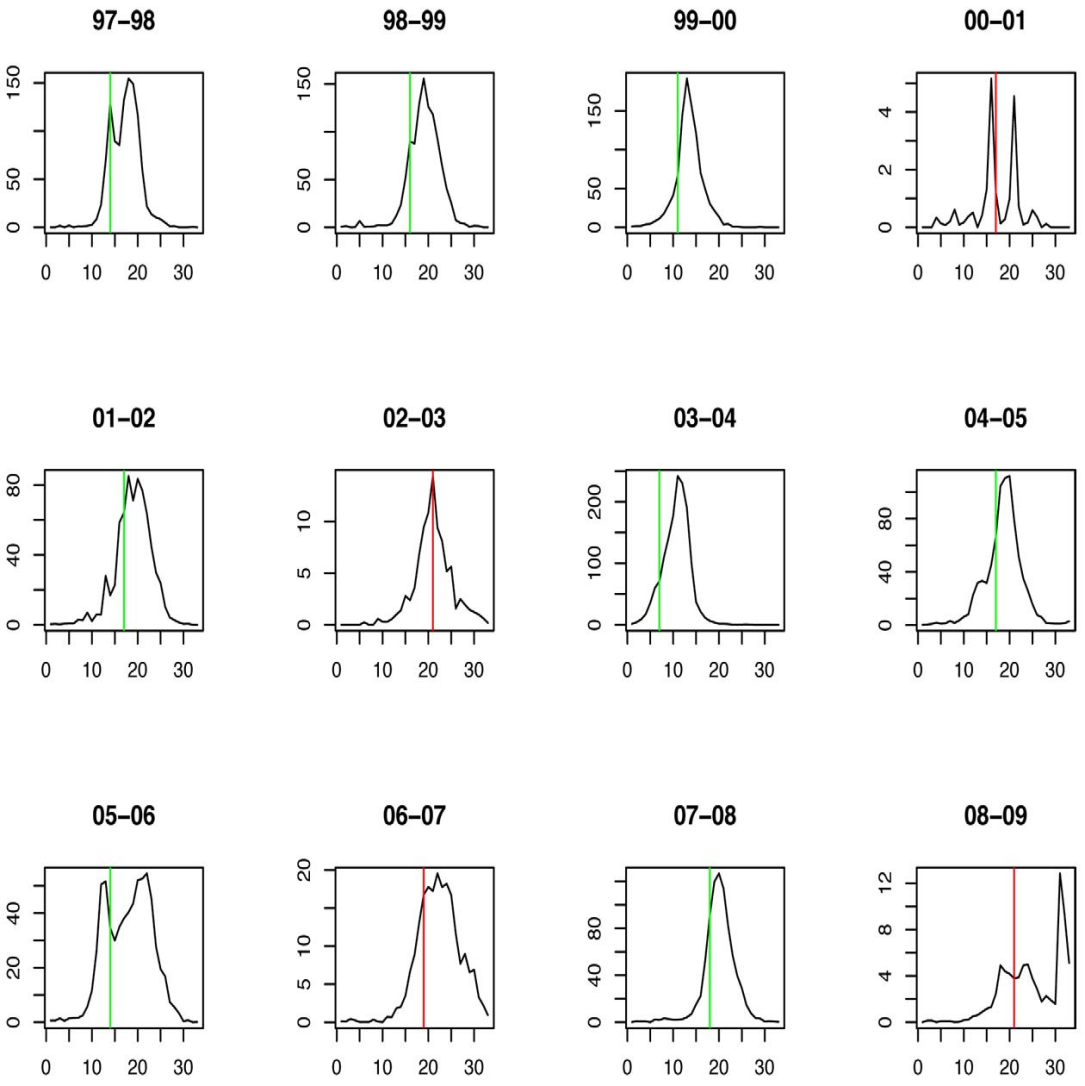
Timing of prediction with regard to weekly influenza A/H3N2 incidence (week 1 = calendar week 40). Green lines show the stopping time *s* in seasons in which the index strain's own threshold was crossed first; red lines indicate the stopping times in seasons in which the complementary threshold was crossed first.

For A/H3N2 the chosen thresholds are *h* = 165, *h*_c_ = 350 (Figures 5 and 6). Prediction occurs before the peak of the A/H3N2 incidence each season except for the (very small) 2000–2001 season.

**Figure 7:**
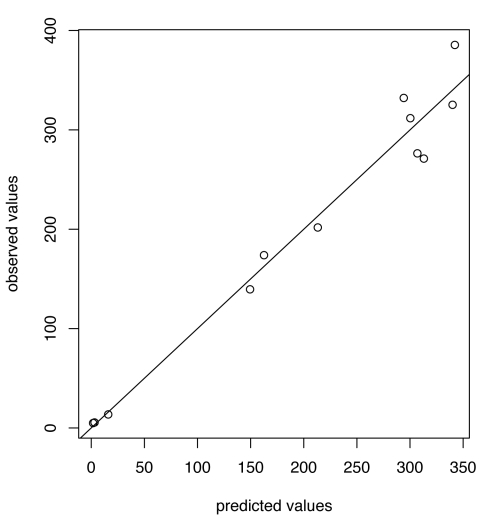
Predicted versus observed values for influenza B for the choice of thresholds *h* = 80, *h*_c_ = 675.

**Figure 8:**
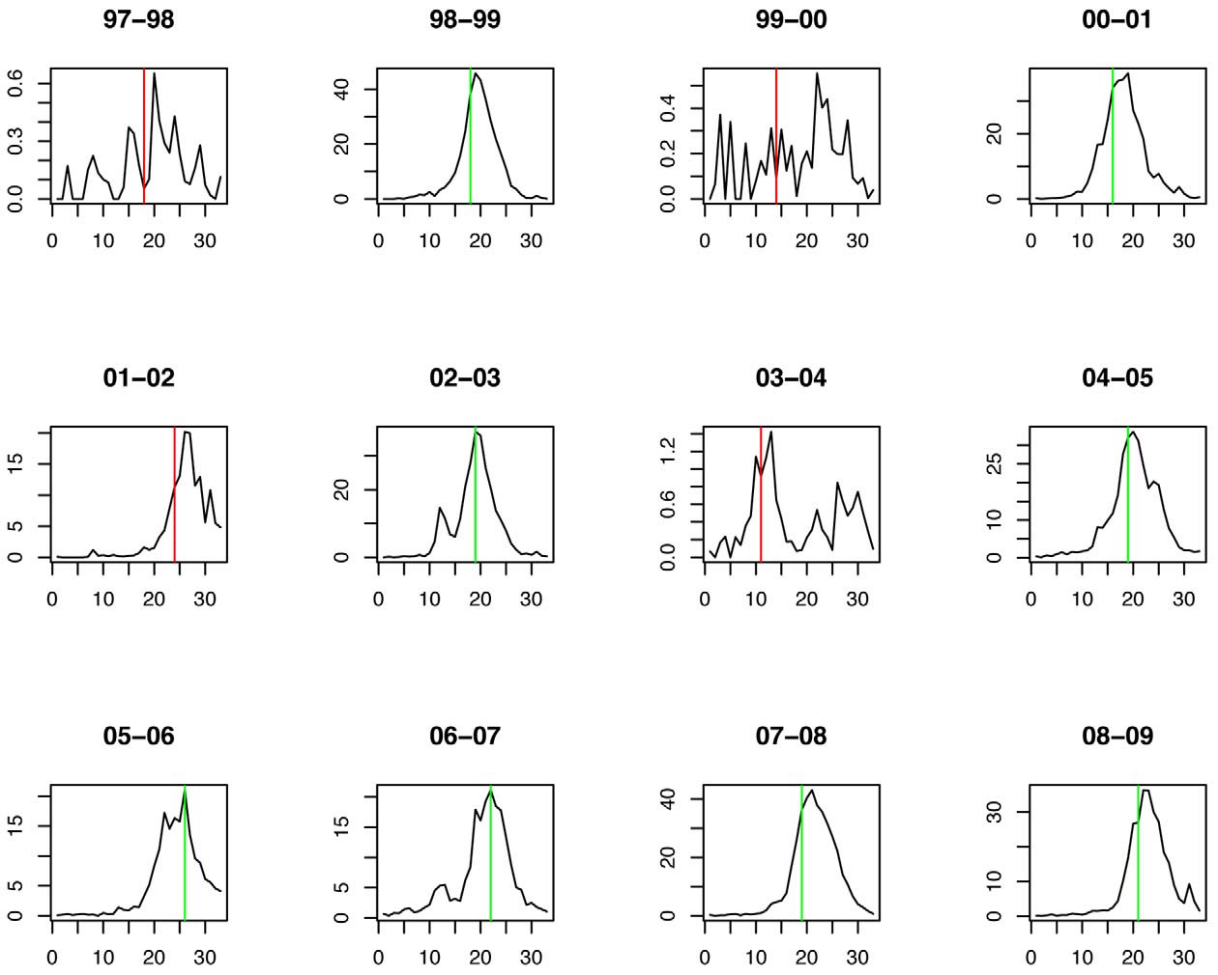
Timing of prediction with regard to weekly influenza B incidence (week 1 = calendar week 40). Green lines show the stopping time *s* in seasons in which the index strain's own threshold was crossed first; red lines indicate the stopping times in seasons in which the complementary threshold was crossed first.

For influenza B the chosen thresholds are *h* = 80, *h*_c_ = 675 (Figures 7 and 8). Prediction occurs before the peak of the B incidence each season.

Table 1 summarizes the estimates and *p*-values for the regression coefficients, as well as the RSE for each model.

**Table 1:** Predictions of the epidemic sizes of A/H1N1 for the thresholds.

Predictions	Coefficient	Coefficient	Coefficient	RSE
**A/H1N1predictions** a				
Quantity	β_X_	β_W_		RSE
Estimate	1,031.65	−9.13		12.02
Standard error	45.8	1.29		
*p*-Value	7·10^−10^	3·10^−5^		
**A/H3N2 predictions** b				
Quantity	β_X_	β_W_	β_0_	RSE
Estimate	1,047.35	−50.43	965	110.1
Standard error	136.45	10.1	209.7	
*p*-Value	3·10^−5^	0.0007	0.0013	
**Influenza B predictions** c				
Quantity	β_X_	β_W_		RSE
Estimate	622.96	−4.13		25.96
Standard error	52.85	1.57		
*p*-Value	3.5·10^−7^	0.025		

a *h* = 140, *h*_c_ = 500.

b Predictions of the epidemic sizes of A/H3N2 for the thresholds *h* = 165, *h*_c_ = 675.

c Predictions of the epidemic sizes of influenza B for the thresholds *h* = 80∼, ∼*h*_c_ = 675.

## Discussion

This study demonstrates how routine virologic and ILI surveillance data can be used to quantify the dynamics of cocirculating influenza strains and generate short-term predictions of the relative epidemic sizes of each strain during the course of an influenza season in the United States. This method should, in principle, be applicable to other settings/regions provided that the datasets are large enough for the estimates of the required quantities (particularly the percent of specimen testing positive for each strain) to be sufficiently robust.

Regional surveillance data on physician visits for ILI and subtyping of virologic specimens were combined to define a proxy for weekly incidence for each of the three strains, A/H3N2, A/H1N1, and B. This incidence proxy revealed for each index strain a negative association between the early CIP of the other two strains (the complementary CIP) and the CIP of the index strain for the whole season, i.e., the strain's epidemic size.

The negative association between strains' incidences suggests that high infection rates with one strain can interfere with the transmission of other strains. As noted above, this correlation may arise from either or both of two mechanisms: early complementary incidence may slow the spread of the index strain, and early, rapid spread of the index strain may slow the spread of the complementary strains. This issue is explored further in Text S1 where we examine the correlation between CIP of various strains during various time periods. Because A/H3N2 is the only strain that had large, early epidemics and showed a negative and significant correlation between its early incidence and the subsequent incidence of the other strains (Text S1), the data most strongly support the idea that A/H3N2 incidence interferes with the circulation of other strains. The negative correlation observed between a high complementary CIP and the epidemic size of A/H3N2 has multiple potential interpretations: the pattern is consistent with the interference of A/H1N1 and/or influenza B with A/H3N2 and the possibility that weak A/H3N2 seasons allowed complementary incidence to grow.

The mechanism of short-term heterologous protection may be also related to a potential impact of vaccination [11]. The population-level impact of vaccination on the proliferation of influenza strains not contained in the vaccine is currently unknown. A Canadian study found that people vaccinated for seasonal influenza had higher attack rates in the subsequent wave of A/H1N1pdm [5], an effect that was likely due at least in part to the lower heterologous protection conferred by the vaccine compared to natural infection. Vaccination against influenza A/H3N2 in mice prevented the induction of hetero-subtypic immunity against avian influenza A/H5N1 virus upon subsequent exposure to influenza A/H3N2 infection [12]. Data in [2] are more ambiguous: higher infection rates with H1N1 pdm for child recipients of seasonal influenza vaccine were recorded (32% versus 17%); however, the statistical significance of this association in the multivariate analysis depends on the adjustment method. One should note that the studies above essentially refer to the impact of the activity of influenza strains contained in the vaccine on the activity of influenza strains not contained in the vaccine, which took place shortly afterwards. The impact of a vaccine on influenza strains cocirculating within the same time period is less clear and further investigations are needed to address this question.

Because seasons with the largest early complementary CIP also had the smallest epidemics of each index strain, we tested the idea that one might be able to predict small seasons of an index strain if the complementary CIP reaches sufficiently high levels in the course of a season. Alternatively, if the index strain's incidence reaches sufficiently high levels first, one might predict the cumulative size of the index strain's incidence from its recent growth rate. We formalize these ideas by introducing thresholds for the complementary CIP and index CIP; reaching either threshold triggers a prediction of the whole-season CIP of the index strain. Because strong seasons of a strain generally start early, this prediction scheme based on threshold crossing should allow for a timely identification of a strain's strong season.

Incidence curves early in the season often have an irregular shape, which might be due to spatial heterogeneity and other factors not accounted for by the model. As a result, the threshold for the index strain's incidence should be high enough and sufficiently conservative so that the growth rate will be an accurate predictor of the epidemic size for that season. Similarly, the threshold for the complementary CIP is needed to be fairly high, potentially reflecting significant interference with the index strain. At the same time, those thresholds shouldn't be set too high or they will be reached either very late in the season (when prediction is of little interest) or never. We expressed the accuracy of the prediction as a function of the two thresholds by the RSE for the historical data [6], and we selected the thresholds in the space of thresholds where the accuracy appeared stable.

Because our model is based on only 12 seasons of observation, it might overfit the data; at the same time, its performance is probably affected by the omission of important factors. True confidence bounds for the prediction should probably be somewhat wider, as suggested by the “leave-one-out” cross-validation (Text S1). One potential source of error is the imperfection of the simple linear model and the predictor estimated from the incidence data. For example, one often sees a dent in the growth patterns of the incidence proxies around the time of winter school closures. If the index strain's threshold, *h*, is crossed in this period, the growth rate at that point might be underestimated. In addition, each choice of thresholds essentially corresponds to a separate model, and we have picked one that gives a good and stable fit to limited existing data. An important direction for future research will be to see if similar predictions can be made by fitting and validating simple models to longer time series.

The definition of the covariate corresponding to the timing of threshold crossing is different for influenza A/H3N2 compared to influenza A/H1N1 and B. The main reason for that difference is that A/H3N2 experienced strong and early seasons in the data. The exceptionally strong and early 2003–2004 season is the only season that has a *p*-value below 0.05 in the leave-one-out cross-validation. However, incorporating this season into the prediction framework should give a better adjustment for the timing of threshold crossing, which in turn should be useful for future predictions of early and strong influenza A/H3N2 seasons. For influenza A/H1N1 and B, calibration of the prediction framework based on the 1997–2009 data might not yield accurate predictions of the sizes of exceptionally strong and early seasons for the index strain. The ongoing, 2010–2011 season contains an early threshold crossing for influenza B compared to the 1997–2009 seasons; moreover, the CIP of influenza B by calendar week 10 of 2011 is already larger than the whole-season CIP of influenza B in any season in the 1997–2009 data. We expect that recalibration of the prediction framework after the current season should give a better adjustment for the timing of threshold crossing for influenza B.

Our method for prediction has several additional limitations that might affect its applicability since the apparent replacement of seasonal A/H1N1 by A/H1N1pdm. The quality of the composite proxy for true influenza infection incidence is uncertain. If the case-reporting rate of A/H1N1pdm differs from the previous seasonal A/H1N1, the predictors might require recalculation. Moreover, the ratio between this indicator and the number of true (serologic) infections may vary by year for each strain. It is unknown how the strength of cross-immunity between the strains depends on strain types and time since infection and it is consequently unknown how A/H1N1pdm might affect the strength of these associations. Answering these questions will require further efforts, including experimental immunological studies and explicit mechanistic models of transmission dynamics (e.g., [S. Cobey et al., personal communication]).

Despite its limitations, this method gives a reasonable prediction of the epidemic sizes of A/H3N2, A/H1N1, and influenza B relative to historical precedents. In particular, the model predicted the relative epidemic size of A/H3N2, the strain associated with the highest rates of mortality [13], on average several weeks before its peak in seasons in which A/H3N2 dominated. Such predictive methods may be useful to decision makers when they are trying to determine in real-time which measures to recommend for an influenza season.

## Supporting Information

Text S1 **Supporting information.**
(DOC)

## Abbreviations

CDC: US Centers for Disease Control
CIP: cumulative incidence proxy
H1N1pdm: pandemic (H1N1) 2009
ILI: influenza-like illness
RSE: residual standard error
