# Supplementary material for: Predicting the Epidemic Sizes of Influenza A/H1N1, A/H3N2, and B: A Statistical Method
Source: PLoS Med. 2011 Jul 5;8(7):e1001051. doi: 10.1371/journal.pmed.1001051 (PMC3130020; doi:10.1371/journal.pmed.1001051)
Supplement: Text S1 — Supporting information. (DOC) [file pmed.1001051.s001.doc]

**Text S1: Supporting Information for “Predicting the epidemic sizes of influenza A/H1N1, A/H3N2 and B”**

Edward Goldstein, Sarah Cobey, Saki Takahashi, Joel C. Miller, Marc Lipsitch

1. Center for Communicable Disease Dynamics, Department of Epidemiology, Harvard School of Public Health, Boston MA, USA
2. Department of Applied Mathematics, Harvard University, Cambridge MA, USA
3. Fogarty International Center, US National Institutes of Health, Bethesda MD, USA
4. Department of Immunology and Infectious Diseases, Harvard School of Public Health, Boston MA, USA

**1. Choice of a calendar week for measuring the association between the complementary cumulative incidence proxy and the epidemic size of each strain**

This section studies the relationship between the cumulative complementary incidence and epidemic size of each of the strains A/H1N1 (Figure S1), A/H3N2 (Figure S2) and influenza B (Figure S3) by calendar weeks 2, 3, 4 and 5 for the 12 seasons in the data. The association was assessed using the Spearman rank (rho) correlation.

- 1. ***Influenza A/H1N1***

**A: Influenza A/H1N1, Epidemiological week 15 (calendar week 2).**

**Spearman rho=** **-0.874, p-value 0.0003**

**B: Influenza A/H1N1, Epidemiological week 16 (calendar week 3)**

**(reproduces main text Figure 2A)**

**Spearman rho=** **-0.874, p-value 0.0003**

**C: Influenza A/H1N1, Epidemiological week 17 (calendar week 4)**

**Spearman rho=** **-0.93, p-value < 0.0000001**

**D: Influenza A/H1N1, Epidemiological week 18 (calendar week 5)**

**Spearman rho=** **-0.93, p-value < 0.0000001**

**Figure S1 (ABCD)** : Relationship between the cumulative incidence of influenza A/H3N2 and B (complementary incidence for A/H1N1) up to epidemiological weeks 15(A),16(B),17(C),18(D) and the cumulative A/H1N1 season during the 12 seasons in the data set.

- 1. ***Influenza A/H3N2***

**A: Influenza A/H3N2, Epidemiological week 15 (calendar week 2)**

**Spearman rho=** **-0.692, p-value= 0.016**

**B: Influenza A/H3N2, Epidemiological week 16 (calendar week 3)**

**(reproduces main text figure 2B)**

**Spearman rho=** **-0.74, p-value= 0.008**

**C: Influenza A/H3N2, Epidemiological week 17 (calendar week 4)**

**Spearman rho=** **-0.755, p-value= 0.007**

**D: Influenza A/H3N2, Epidemiological week 18 (calendar week 5)**

**Spearman rho=** **-0.755, p-value= 0.007**

**Figure S2 (ABCD)** : Relationship between the cumulative incidence of influenza A/H1N1 and B (complementary incidence for A/H3N2) up to epidemiological weeks 15(A),16(B),17(C),18(D) and the cumulative A/H3N2 season during the 12 seasons in the data set.

- 1. ***Influenza B***

**A: Influenza B, Epidemiological week 15 (calendar week 2)**

**Spearman rho=** **-0.615, p-value= 0.037**

**B: Influenza B, Epidemiological week 16 (calendar week 3)**

**(reproduces main text Figure 2C)**

**Spearman rho=** **-0.55, p-value= 0.066**

**C: Influenza B, Epidemiological week 17 (calendar week 4)**

**Spearman rho=** **-0.52, p-value= 0.089**

**D: Influenza B, Epidemiological week 18 (calendar week 5)**

**Spearman rho=** **-0.256, p-value= 0.36**

**Figure S3 (ABCD)** : Relationship between the cumulative incidence of influenza A/H1N1 and A/H3N2 (complementary incidence for B) up to epidemiological weeks 15(A),16(B),17(C),18(D) and the cumulative B season during the 12 seasons in the data set.

**2. Correlation between the complementary cumulative incidence up to a given week and the index strain’s cumulative incidence after this week**

As noted in the main text, if the circulation of each strain interferes with the others, then a negative correlation between their cumulative incidences could arise by two mechanisms. First, a high complementary CIP would slow the spread of the index strain. Second, large seasons of an index strain may begin early, interfering with the spread of the complementary strains by the chosen calendar week. Here we attempt to separate these mechanisms by studying for each strain the correlation between the complementary CIP up to a calendar week $x$ and the strain’s cumulative incidence starting in calendar week $x+1$. This correlation, compared to the observed relationship between the complementary CIP and the whole-season CIP (Figure 2), should reflect primarily the impact of the complementary strains on the index strain. Figure S4 shows the results for the three strains for epidemiological week 16.

**A: Influenza A/H1N1, Spearman rho -0.895, p-value <0.00001**

**B: Influenza A/H3N2, Spearman rho -0.203, p-value 0.53**

**C: Influenza B, Spearman rho -0.608 , p-value 0.04**

**Figure S4(ABC):** The complementary CIP up to epidemiological week 16 and the index strain’s cumulative incidence starting in epidemiological week 17 for (A) A/H1N1. (B) A/H3N2 and (C) Influenza B.

For influenza A/H1N1 and B, the negative correlation observed is even stronger than the observed relationship between the complementary CIP up to week 16 and the whole-season CIP of the index strain (Figure 2). This strengthening of the correlation when considering only the later incidence of the index strain is consistent with complementary incidence interfering with the incidence of either A/H1N1 or B. For A/H3N2, in contrast, the correlation becomes much weaker. Some of the strongest seasons on the left of Figure S2B have a much smaller incidence starting week 17, making the overall correlation in Figure S4B small and statistically insignificant. Thus, the evidence from these correlations for interference by A/H1N1 and B on the circulation of A/H3N2 is less compelling than that for A/H3N2’s interference with the circulation of A/H1N1 and B.

Table S1 shows the temporal progression of the correlation between complementary CIP up to a calendar week x and the cumulative incidence of the index strain starting in calendar week x+1 for each of the strains.

***(A***) Influenza H1N1

| Week | 8 | 10 | 12 | 14 | 16 | 18 |
| --- | --- | --- | --- | --- | --- | --- |
| Spearman  rho | -.67 | -.67 | -.82 | -.88 | -.895 | -.937 |
| p-value | .02 | .02 | .002 | .00019 | .000006 | <2*10 |

(B) Influenza H3N2

| Week | 8 | 10 | 12 | 14 | 16 | 18 |
| --- | --- | --- | --- | --- | --- | --- |
| Spearman  Rho | -.61 | -.6 | -.51 | -.38 | -.2 | -.06 |
| p-value | .04 | .043 | .094 | .218 | .528 | .85 |

(A) Influenza B

| Week | 8 | 10 | 12 | 14 | 16 | 18 |
| --- | --- | --- | --- | --- | --- | --- |
| Spearman  rho | -.077 | -.19 | -.38 | -.7 | -.61 | -.45 |
| p-value | .82 | .56 | .23 | .015 | .04 | .15 |

**Table S1(ABC):** Correlation between the complementary CIP up to week $x$ and the cumulative incidence of the index strain starting in week $x+1$ for (A) A/H1N1, (B) A/H3N2 and (C) Influenza B.

**3. Correlation between strains’ whole-season CIPs**

Table S2 records the whole-season CIP of each strain and the whole-season CIP of all strains for the 12 seasons in the data.

| Strain/  season | 97-98 | 98-99 | 99-00 | 00-01 | 01-02 | 02-03 | 03-04 | 04-05 | 05-06 | 06-07 | 07-08 | 08-09 |
| --- | --- | --- | --- | --- | --- | --- | --- | --- | --- | --- | --- | --- |
| A/H3N2 | 1087 | 1056 | 996 | 18.6 | 718 | 93.4 | 1473 | 816 | 663 | 211 | 809 | 51.5 |
| A/H1N1 | 1.7 | 5.9 | 28.2 | 386 | 37.9 | 276 | 0.13 | 2.6 | 60.7 | 491 | 249 | 442.6 |
| B | 5 | 325.4 | 5.6 | 332.2 | 139.6 | 271.2 | 13.7 | 311.9 | 174 | 201.9 | 385.6 | 276.5 |
| Overall | 1094 | 1388 | 1030 | 737 | 895 | 641 | 1487 | 1131 | 898 | 904 | 1444 | 771 |

**Table S2:** Whole-season CIP of each strain and the total CIP of all strains for each season.

We also examined the correlation between the complementary whole-season CIP and the index strain’s whole-season CIP for each of the three strains.

**A: Influenza A/H1N1, Spearman rho -0.811, p-value 0.0024**

**B: Influenza A/H3N2, Spearman rho -0.811, p-value 0.0024**

**C: Influenza B, Spearman rho -0.329, p-value 0.297**

**Figure S5(ABC):** Whole-season complementary CIP and the whole-season CIP of the index strain for (A) A/H1N1, (B) A/H3N2 and (C) influenza B.

**4. Pairwise correlations**

We further explored the potential interference in the strains’ dynamics by exploring the pairwise correlations between the CIP of some strain $S_{1}$ up to week $x$ and the cumulative incidence of another strain $S_{2}$ starting in week $x+1$. The correlation was calculated for each ordered pair of strains.

| Week | 8 | 10 | 12 | 14 | 16 | 18 |
| --- | --- | --- | --- | --- | --- | --- |
| Spearman rho | -.76 | -.73 | -.79 | -.85 | -.867 | -.867 |
| p-value | .006 | .01 | .0036 | .0008 | .0004 | .0004 |

**Table S3.1:** Correlation between the CIP of H3N2 up to week x and the cumulative incidence of A/H1N1 starting week x+1

| Week | 8 | 10 | 12 | 14 | 16 | 18 |
| --- | --- | --- | --- | --- | --- | --- |
| Spearman rho | -.64 | -.71 | -.57 | -.46 | -.32 | -.29 |
| p-value | .023 | .01 | .055 | .13 | .3 | .035 |

**Table S3.2:** Correlation between the CIP of A/H1N1 up to week x and the cumulative incidence of A/H3N2 starting week x+1

| Week | 8 | 10 | 12 | 14 | 16 | 18 |
| --- | --- | --- | --- | --- | --- | --- |
| Spearman rho | -.22 | -.38 | -.56 | -.64 | -.58 | -.497 |
| p-value | .5 | .22 | .06 | .03 | .05 | .1 |

**Table S3.3:** Correlation between the CIP of A/H3N2 up to week x and the cumulative incidence of influenza B starting week x+1

| Week | 8 | 10 | 12 | 14 | 16 | 18 |
| --- | --- | --- | --- | --- | --- | --- |
| Spearman rho | -.45 | -.447 | -.52 | -34 | -.196 | .008 |
| p-value | .14 | .147 | .08 | .287 | .54 | .8 |

**Table S3.4:** Correlation between the CIP of influenza B up to week x and the cumulative incidence of A/H3N2 starting week x+1

| Week | 8 | 10 | 12 | 14 | 16 | 18 |
| --- | --- | --- | --- | --- | --- | --- |
| Spearman rho | .5 | .47 | .52 | .448 | .39 | .35 |
| p-value | .096 | .12 | .08 | .14 | .2 | .26 |

**Table S3.5:** Correlation between the CIP of A/H1N1 up to week x and the cumulative incidence of influenza B starting week x+1

| Week | 8 | 10 | 12 | 14 | 16 | 18 |
| --- | --- | --- | --- | --- | --- | --- |
| Spearman rho | .53 | .4 | .426 | .377 | .25 | .21 |
| p-value | .07 | .21 | .165 | .22 | .42 | .51 |

**Table S3.6:** Correlation between the CIP of influenza B up to week x and the cumulative incidence of A/H1N1 starting week x+1

Given that A/H3N2 is essentially the only strain that had large, early epidemics and a consistently negative and significant correlation between its early incidence and the subsequent incidence of the other strains, the data are most consistent with the idea that A/H3N2 interferes with the circulation of other strains. The positive and insignificant correlations for A/H1N1 and B may be confounded by the fact that strong, early A/H3N2 epidemics lead to weak A/H1N1 and B epidemics, leading to an indirect association between weak A/H1N1 seasons and weak B seasons.

**5. Choice of thresholds**

In this section, we examined the accuracy and timeliness of the prediction for the various choices of thresholds as described in the Methods. The accuracy was measured by the residual standard error for the prediction (Figure S6); the timeliness was measured by the mean time between the week when incidence for the chosen strain has peaked and the prediction week (Figure S7, negative values correspond to a more timely prediction).

***5.1 Accuracy of predictions***

Figure S6 depicts the residual standard error (RSE) of the prediction for each strain as the function of the strain’s own threshold $h$and the complementary threshold $h_{c}$.

If neither threshold was reached during a season, the predictors were set to $0$. The outcome was still defined as the cumulative incidence of the index strain. (This situation arose in some regions of the parameter space of thresholds but not for the thresholds used in the main text).

**A: Influenza B, residual standard errors for prediction**

**B: Influenza A/H1N1, residual standard errors for prediction**

**C: Influenza A/H3N2, residual standard errors for prediction**

**Figure S6(ABC):** RSE for prediction for various choices of own and complementary thresholds for the three index strains. (A) – Influenza B, (B) – Influenza A/H1N1, (C) – Influenza A/H3N2.

***5.2 Timeliness of prediction***

The average timeliness of prediction was measured as the mean of (prediction week – peak weak) for each of the index strains. Negative timeliness indicates that predictions on average were made before the peak of the index strain’s incidence. Figure S7 plots the average timeliness of prediction as a function of the thresholds used for prediction.

**A: Influenza B, timeliness of prediction**

**B: Influenza A/H1N1, timeliness of prediction**

**C: Influenza A/H3N2, timeliness of prediction**

**Figure S7(ABC):** The timeliness of prediction (mean of (prediction week – peak week)) for each of the influenza strains as a function of thresholds $h$ and $h_{c}$. (A) Influenza B, (B) A/H1N1, (C) A/H3N2.

**6. “Leave-one-out” cross validation for prediction results**

For each of the index strains, we performed “leave-one-out” cross validation for the predictions using the thresholds from the Results section. Specifically, for each season, we removed the data on the covariates and the outcome during that season and performed the regression analysis with the remaining covariates and outcomes. After computing the regression coefficients, we reincorporated the result for the omitted season to compute the (two-sided) p-value for the corresponding t-statistic arising from the difference between the prediction and the outcome for the omitted season . Table S4 gives those p-values for each index strain and omitted season.

| Strain/season | 97-98 | 98-99 | 99-00 | 00-01 | 01-02 | 02-03 | 03-04 | 04-05 | 05-06 | 06-07 | 07-08 | 08-09 |
| --- | --- | --- | --- | --- | --- | --- | --- | --- | --- | --- | --- | --- |
| A/H1N1 | .59 | .91 | .91 | .99 | .1 | .7 | .99 | .97 | .003 | .44 | .94 | .79 |
| A/H3N2 | .55 | .44 | .36 | .16 | .55 | .8 | .023 | .17 | .3 | .89 | .63 | .33 |
| B | .9 | .56 | .93 | .12 | .69 | .08 | .93 | .68 | .52 | .64 | .06 | .22 |

**Table S4:** p-values for the “leave-one-out” cross validation

For influenza B, no outliers exist in the leave-one-out cross validation for the 12 seasons in the data. For A/H3N2, the exceptionally strong and early 2003-2004 season is the only season which has a p-value below .05 in the leave-one-out cross validation. However incorporating this season into the prediction framework should give a better adjustment for the timing of threshold crossing, which in turn should be useful for future predictions of early and strong influenza A/H3N2 seasons. For A/H1N1, prediction gives a very close fit to the observed values for most seasons in the data (Figure 3A). For the 2005-2006 season, the fit is less close and this season is an outlier in the leave one out cross validation. Incorporating this season into the prediction framework increases the confidence bounds for the prediction somewhat. Still, prediction accuracy is very high for a sufficiently wide region in the space of thresholds for A/H1N1 (Figure S6B), and the usage of those thresholds presumably has some degree of over-fitting.

**7. Predicting the 2010-2011 season**

We used data from up to calendar week 10 of 2011 (epidemiological week 23) to make predictions for the ongoing 2010-2011 season. Figure S8 represents the weekly incidence proxies for A/H3N2, A/H1N1 and influenza B.

**Figure S8:** Weekly incidence of influenza A/H3N2 (green), A/H1N1 (blue) and B (red) strains, 2010-2011 season.

Table S5 presents the prediction results as well as the cumulative incidence up to week 23 for each strain.

| Strain | Week/type of threshold crossing | Prediction | Cumulative incidence up to week 23 |
| --- | --- | --- | --- |
| A/H1N1 | 17 (complementary) | 470.7 | 424.5 |
| A/H3N2 | 14 (own) | 664.3 | 679.7 |
| B | 13 (own) | 319 | 390.4 |

**Table S5:** Prediction results for the 2010-2011 season

For A/H1N1, prediction appears to be reasonable given the change in incidence up to week 23. For A/H3N2, the prediction was made on week 15, after the decline in the growth of incidence, which coincided with winter school closures. As a result, the growth rate proxy on week 15 appears to underestimate somewhat the cumulative season size, though the actual size should be safely within the confidence bounds for the prediction. Influenza B crossed its own threshold on week 13, considerably earlier than such crossings in the historical data. (Between the 1997-1998 and 2008-2009 seasons, the earliest influenza B crossed its own threshold was week 16 in the 2000-2001 season.) Moreover, the threshold in this season was crossed in the last week of 2010. While adjusting for the timing of threshold crossing raises the estimate of the epidemic size of influenza B somewhat compared to the prediction using the covariate $X$alone (Figure S10), the resulting estimate should be significantly lower than the actual incidence (which by week 23 is larger than the epidemic size for each of the seasons in the 1997-2009 data). Part of the reason for a low estimate may be due to the fact that prediction was calibrated against data that had no such large and early influenza B seasons. Re-calibrating the prediction framework for influenza B after the current season is advisable for future predictions.

**8. Alternative predictive models**

Our prediction framework estimates the epidemic size $Y$ of an index strain linearly in terms of the two predictors: the recent growth proxy $X$and the (weighted) time $T$ of crossing the threshold. Initially, we attempted to predict $Y$in terms of $X$alone, which in principle would be possible if the real dynamics followed a mass-action model with a low reproductive number (close to 1) where linearization is a reasonable approximation for the quantities involved. However, due to seasonality in influenza transmission, the timing of threshold crossing affects the growth rate at that time. As explained in the Methods, the 2003-2004 season for A/H3N2 was an outlier for the prediction scheme using the predictor $X$alone. For completeness, we describe the alternative, simpler model here in the context of the two strains for which it is appropriate.

For A/H1N1 and B, prediction using $X$alone could be carried through a prediction scheme similar to the one described in the main body of the text. For each index strain (A/H1N1 or B), the outcome $Y$ was predicted linearly in terms of $X$, with the intercept not being statistically significant for the 1997-2009 data:

$Y=\beta\cdot X$ (S1)

We chose the thresholds $h$and $h_{c}$ for each strain by visually inspecting the RSEs for a wide range of thresholds (Figure S9A and S10A) and determining the space of thresholds where RSE is low and stable. Predictions for a choice of thresholds are shown for A/H1N1 (Figure S9B) and influenza B (Figure S10B).

**(A) A/H1N1, Residual standard errors for prediction**

**(B) A/H1N1, Prediction line and confidence bounds**

**Figure S9(AB):** Prediction results for A/H1N1 using equation (S1). (A) Residual standard errors for prediction. (B) Prediction line and 95% confidence bounds for the choice of thresholds $h=140,h_{c}=500$

**(A) Influenza B, Residual standard errors for prediction**

**(B) Influenza B, Prediction line and confidence bounds**

**Figure S10(AB):** Prediction results for influenza B using equation (S1). (A) Residual standard errors for prediction. (B) Prediction line and 95% confidence bounds for the choice of thresholds $h=75,h_{c}=675$

The definition of the covariate $T$ is different for A/H3N2 compared to A/H1N1 and B. If one defines $T$for A/H1N1 or B in the same manner as for A/H3N2, the covariate $T$ would be positively correlated with the outcome $Y$for strong and early A/H3N2 seasons, whereas $T$ is negatively correlated with $Y$for strong seasons of the index strain (either A/H1N1 or B). The resulting model for A/H1N1 or B would have worse fits than the main model we are using and, more importantly, the covariate $T$would not be statistically significant in that model. Vice versa, if one defines the covariate $T$for A/H3N2 in the same manner as we did for A/H1N1 and B, it would not be statistically significant in the model, and moreover the 2003-2004 season would persist as an outlier.

**References**
